# The Role of lncRNAs in the Pathobiology and Clinical Behavior of Multiple Myeloma

**DOI:** 10.3390/cancers13081976

**Published:** 2021-04-20

**Authors:** Arantxa Carrasco-León, Ane Amundarain, Nahia Gómez-Echarte, Felipe Prósper, Xabier Agirre

**Affiliations:** 1 Área de Hemato-Oncología, Centro de Investigación Médica Aplicada (CIMA), IDISNA, Universidad de Navarra, Avenida Pío XII-55, 31008 Pamplona, Spain; acleon@alumni.unav.es (A.C.-L.); aamundarain.1@alumni.unav.es (A.A.); ngomez.9@alumni.unav.es (N.G.-E.); 2 Centro de Investigación Biomédica en Red de Cáncer (CIBERONC), 31008 Pamplona, Spain; 3 Departamento de Hematología, Clínica Universidad de Navarra, Universidad de Navarra, Avenida Pío XII-36, 31008 Pamplona, Spain

**Keywords:** lncRNAs, multiple myeloma, RNA-based therapy

## Abstract

**Simple Summary:**

Multiple myeloma (MM), the second most common hematological neoplasm, is still considered an incurable disease. Long non-coding RNAs (lncRNAs), genes that do not encode proteins, participate in numerous biological processes, but their deregulation, like that of coding genes, can contribute to carcinogenesis. Increasing evidence points to the relevant role of lncRNAs in the development of human tumors, such that they emerge as attractive biomarkers and therapeutic targets for cancer treatment, including MM. Here we review the oncogenic or tumor-suppressor functions of lncRNAs in MM and provide an overview of novel therapeutic approaches based on lncRNAs that will help to improve the management of these patients.

**Abstract:**

MM is a hematological neoplasm that is still considered an incurable disease. Besides established genetic alterations, recent studies have shown that MM pathogenesis is also characterized by epigenetic aberrations, such as the gain of de novo active chromatin marks in promoter and enhancer regions and extensive DNA hypomethylation of intergenic regions, highlighting the relevance of these non-coding genomic regions. A recent study described how long non-coding RNAs (lncRNAs) correspond to 82% of the MM transcriptome and an increasing number of studies have demonstrated the importance of deregulation of lncRNAs in MM. In this review we focus on the deregulated lncRNAs in MM, including their biological or functional mechanisms, their role as biomarkers to improve the prognosis and monitoring of MM patients, and their participation in drug resistance. Furthermore, we also discuss the evidence supporting the role of lncRNAs as therapeutic targets through different novel RNA-based strategies.

## 1. Introduction

Multiple myeloma (MM) is a hematological neoplasm characterized by the uncontrolled aberrant clonal proliferation of plasma cells (PCs) in the bone marrow [1]. This disease is the second most common hematological malignancy, after non-Hodgkin lymphoma [2,3], affecting elderly patients with a median age of 65 years [4]. Despite the latest advances in treatment strategies, which have significantly increased patient survival, MM is still considered an incurable disease, with a median overall survival of 7 years.

MM is a very heterogeneous disease, which is reflected in the inter-individual differential diagnosis and survival of patients. Different studies have associated this variability with a wide range of genetic and epigenetic alterations present in MM patients [5,6], including distinct molecularly defined subtypes with different features [7]. Regarding the genetic variability, MM is divided into hyperdiploid (HRD) and non-HRD subtypes [7]. HRD MM is characterized by the trisomy of chromosomes 3, 5, 7, 9, 11, 15, 19 and 21 [6], whereas non-HRD MM is characterized by translocations of the immunoglobulin (Ig) alleles. The majority of these translocations affect chromosome 14, where the Ig H-chain is located [6,7]. However, Ig translocations can also affect the kappa and lambda light chains, the co-occurrence of which is common with HRD MM. Besides, some of these light-chain translocations are associated with a poor outcome for MM patients, as is the case for IgL-MYC translocations [8]. Some of the common heavy-chain translocations are also considered as high-risk prognostic factors, such as t(4;14) and t(14;16), which affect *MMSET* and *MAF* genes, respectively [6,7,9]. Epigenetic aberrations of the DNA methylation and histone modifications are also thought to play an important role in MM pathogenesis. The study of global DNA methylation of MM has led to the identification of a highly heterogeneous DNA methylation pattern, which results in extensive DNA hypomethylation of intergenic regions and DNA hypermethylation associated with intronic and enhancer regions [2,5]. In addition, the study of histone modifications in MM has revealed a de novo gain of active chromatin marks preferentially located in regulatory elements, such as enhancer and promoter regions, which arise from heterochromatic regions in normal B cells [10,11,12]. These results suggest the possibility that these epigenetically regulated non-coding genomic regions could lead to the transcription of non-coding RNA genes (ncRNAs) and, in particular, to the expression of long non-coding RNAs (lncRNAs), which may play a relevant role in the pathobiology and clinical outcome of MM [13]. Nowadays, studies about the role of certain lncRNAs in MM are emerging. However, more comprehensive analyses are required to better understand their function in this disease. In this review, we summarize the current knowledge regarding the role of lncRNAs in the development and outcome of MM and discuss the possibility of lncRNAs as targets for the development of novel RNA-based therapeutic strategies for MM patients.

## 2. Features of lncRNAs

Traditionally, cellular functions of DNA and proteins have overshadowed the roles of RNAs. In recent years, the development of high-throughput techniques, such as RNA sequencing (RNA-seq), has brought great advances in the understanding of the cell transcriptome. So far, it is known that, although only 1–2% of the human genome is translated into proteins, around 70–90% of it is transcribed into RNA, resulting in a huge amount of ncRNAs [14]. Among these ncRNA genes, lncRNAs are defined as those non-coding transcripts longer than 200 nt that do not encode proteins, with open reading frames (ORFs) smaller than 100 amino acids, and with a lack of or low coding potential. However, the latest RNA-seq studies have shown that some lncRNAs contain cryptic ORFs, which could encode for small ORFs or non-conserved peptides [15,16,17].

**Figure 1:**
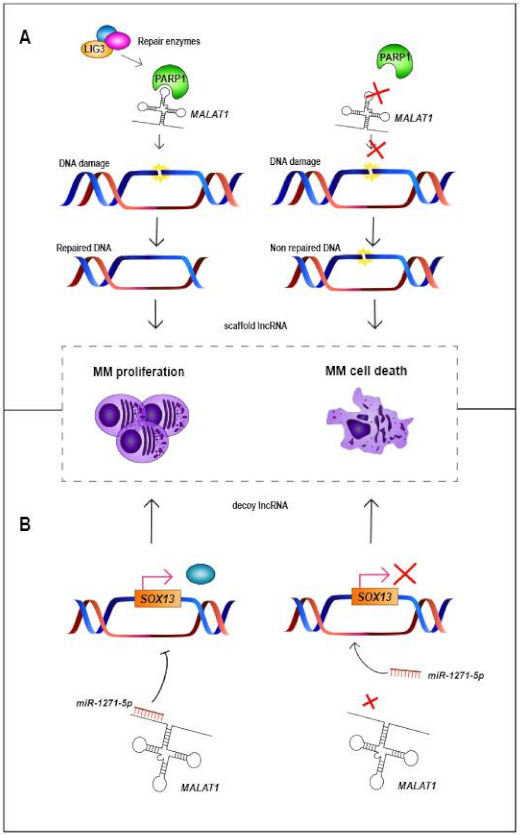
Mechanisms by which *MALAT1* acts in MM cells. (**A**) *MALAT1* acts as scaffold lncRNA, binding to PARP1 protein, which binds to a complex of DNA-repair enzymes consisting of LIG3 among others. Then, the protein–*MALAT1* complex repairs the damaged DNA, triggering the proliferation of MM cells. However, when binding of *MALAT1* and PARP1 does not occur, damaged DNA is not repaired, triggering MM cell death [70]. (**B**) *MALAT1* can also act as a miRNA sponge (decoy), binding to different miRNAs such as *miR-1271-5p*, a tumor-suppressor miRNA that negatively regulates SOX13. Binding of *MALAT1* and *miR-1271-5p* triggers overexpression of SOX13 and proliferation of MM cells, whereas knockdown of *MALAT1* releases *miR-1271-5p*, which binds and prevents translation of SOX13. MM = multiple myeloma.

The characteristics of lncRNAs may differ from each other and they can be capped at the 5′end, spliced and/or polyadenylated (poly(A)+). Remarkably, transcripts with the poly(A)+ tail have higher stability than those with poor or no polyadenylation. On the other hand, there are lncRNAs that can present both polyadenylated and non-polyadenylated isoforms, such as *MALAT1* (metastasis associated lung adenocarcinoma transcript 1) or *NEAT1* (nuclear paraspeckle assembly transcript 1) [18,19]. Although the size of lncRNAs varies between 200 nt and more than 1 MB (known as macro lncRNAs), 42% of lncRNAs only present two exons [19,20]. In contrast to mRNAs, which are located at the cytosol, lncRNAs can be located either in the nucleus or in the cytoplasm, where they can exert various functions. Thus, regarding the location where lncRNAs act and their transcription site, they are capable of acting as *cis* and/or *trans* transcripts [15,21]. *Cis* lncRNAs are known to influence the expression and/or chromatin states of their neighboring genes, while *trans* lncRNAs act over distal genes [22,23,24]. Interestingly, lncRNAs are cell- and tissue-specific, and they may affect different biological processes, such as chromosome conformation, imprinting of genomic loci, or gene and protein regulation [15,25]. lncRNAs have the ability to regulate at DNA, RNA and protein levels, and their functions can be divided into four different groups depending on their molecular mechanisms [18,26]: (1) signal lncRNAs are regulatory molecules that can trigger the transcription of other genes by their presence. They can infer chromatin states, affect gene imprinting or mark certain spaces, times or stages for gene regulation, such as *Air* or *PANDA* (p21-associated ncRNA DNA damage activated) [26,27]. (2) Decoy lncRNAs are transcripts that bind to targets and prevent them from binding to their own targets, thus leading to the alteration of post-transcriptional control. This type of lncRNA can act as an miRNA sponge, binding to miRNAs thanks to their complementary sequence (Figure 1) [26]; *PTENP1* (phosphatase and tensin homolog pseudogene 1), for example, leads to tumor suppressor activity due to the decoy of different miRNAs [28,29,30]. (3) Guide lncRNAs can regulate gene expression through the recruitment and re-localization of ribonucleoprotein complexes at specific chromatin loci, such as *MEG3* (maternally expressed 3), which guides the EZH2 subunit to TGFβ-regulated genes (Table 1) [18,31,32]. (4) Scaffold lncRNAs can act as central platforms upon the assembly of different ribonucleoprotein complexes, affecting their molecular components (Figure 1) [32]; for instance, *HOTAIR* (HOX transcript antisense intergenic RNA) adopts a four-module secondary structure for the interaction with polycomb repressive complex 2 (PRC2) (Table 1), promoting gene repression [18,26].

## 3. Role of lncRNAs in the Pathobiology of MM

Diverse studies have pointed to the importance of lncRNAs in different biological processes, such as immune response, cell differentiation, gene expression modulation and chromatin reorganization [98,99]. Intriguingly, their deregulation also contributes to the development of carcinogenesis, metastasis and even anti-cancer treatment resistance [63]. The deregulation of the expression of lncRNAs can thus impact on relevant pathways involved in the pathogenesis and/or progression of certain human tumors, including MM [43,71]. We have recently demonstrated that 82% of the transcriptome, including coding genes and all types of polyA+ and non-polyA lncRNAs, in plasma cells from MM correspond to lncRNAs, compared to 18% of coding genes [13].

Some deregulated lncRNAs in MM also appear deregulated in the same way in other types of human cancer: for example, *HOTAIR* is upregulated in hepatocellular carcinoma (HCC), *PDIA3P* (protein disulfide isomerase family A member 3 pseudogene 1) in lung cancer, and *LINC00461* in both HCC and lung cancer, all three of them being also upregulated in MM [47,48,49,54,55,56,100]. *PRAL* (P53 regulation associated lncRNA) is downregulated in HCC, lung cancer and MM, and *GAS5* (growth arrest specific 5) in breast, prostate, renal cancer and MM [43,86,87,101,102]. However, there are lncRNAs that are deregulated in MM while they show the opposite direction of expression in other neoplasms. For instance, *MALAT1* is upregulated in MM, lung cancer, gallbladder cancer, colorectal carcinoma and HCC, whilst this lncRNA is downregulated in colorectal and glioma cancer [64,103,104]. *NEAT1* is also upregulated in MM, lung cancer and HCC, but is downregulated in acute promyelocytic leukemia [105]. Finally, *Circ_0000190* is downregulated in MM and gastric cancer, whereas it displays overexpression in lung cancer [36,37,38]. These results highlight the cell- and tissue- specificity of lncRNAs, showing that their deregulation—and thus, their potential function—needs to be addressed in each tumor. For example, *MALAT1* (one of the most widely studied lncRNAs [103]) and *NEAT1* are able to bind or interfere with different molecules and pathways depending on the tissue or disease (Table 1) [77,106]. *MALAT1* acts as an miRNA sponge binding to *miR-1271-5p* (Figure 1), *miR-181a-5p* and *miR-509-5p* in MM, to *miR-195* in HCC or to *miR-206* and miR-363-3p in gallbladder cancer [63,65,66,67,68]. In the case of *NEAT1*, it binds to *miR-125a* in MM and to *miR-193a-3p* in lung adenocarcinoma, among others [77,106]. Usually, the expression of lncRNAs and miRNAs is negatively correlated. Therefore, overexpression of one lncRNA could trigger the downregulation of miRNAs, whereas downregulation of one lncRNA could promote the overexpression of different miRNAs [33,77]. Likewise, there are other examples of lncRNAs which act as miRNA sponges in MM (Table 1). In MM, some of these lncRNAs, such as *CRNDE* (colorectal neoplasia differentially expressed) and *IRAIN* (IGF1R antisense imprinted non-protein coding RNA) are associated with the regulation of one single miRNA. However, an increasing number of studies in MM are showing that lncRNAs can regulate or can be regulated by more than one miRNA, such as *H19* (H19 imprinted maternally expressed transcript), *UCA1* (urothelial cancer associated 1) or *OIP5-AS1* (OIP5 antisense RNA 1). Remarkably, there are cases like *TUG1* (taurine up-regulated 1) and *H19* that are associated with the regulation of the same miRNA, *miR-29b-3p* (Table 1) [33,39,42,44,45,51,53,57,58,59,60,72,81,82,83,88,90,91,94,95,96]. These results highlight the relevance of the miRNA sponge function of lncRNAs in MM.

Different studies have revealed how the knockdown or upregulation of certain lncRNAs is also associated with different biological and phenotypic effects in MM cells, such as the decrease in cell proliferation or viability, the decrease in cellular migration, the increase in cellular apoptosis and cell cycle arrest (Table 1).

Furthermore, various studies have demonstrated the in vivo biological effect of lncRNA knockdown in MM. For example, the inhibition of *DARS-AS1* (DARS antisense RNA 1) or *LINC00152* reduces the tumorigenesis of MM cells, whilst the knockdown of *SOX2OT* (SOX2 overlapping transcript) reduces tumor growth. Moreover, the knockdown of *LINC01234* increases *miR-124-3p* and suppresses *GRB2* expression, resulting in a decrease of cell proliferation and the inhibition of MM growth. These results demonstrate that lncRNAs play an important role in the pathobiology of MM (Table 1) [13,33,34,35,39,40,41,42,43,44,45,48,50,51,53,56,57,58,59,62,69,72,78,79,81,82,83,84,85,86,88,90,91,92,94,95,96,97,107].

## 4. Impact of lncRNAs on the Response of MM

As mentioned above, some lncRNAs interfere with the clinical response of MM patients to different drugs used for their treatment (Table 1). The knockdown of *HOTAIR* expression in MM cells triggers a decrease in chemoresistance to drugs such as dexamethasone, and the silencing of *MIAT* (myocardial infarction associated transcript) and *PCAT-1* (prostate cancer associated transcript 1) sensitized MM cells to bortezomib [43,48,49,75,76,83,84]. It is interesting how *HOTAIR* and *PCAT-1* are associated with PRC2 epigenetic complex in other neoplasms. These two lncRNAs interact with PRC2, acting as epigenetic repressors of chromatin and inducing the reprogramming of genome chromatin states, or modulating gene transcription, respectively. Thus, it is possible that more lncRNAs and other transcripts that act by regulating or interfering with PRC2 could be also associated with resistance or sensitization to the treatment of MM patients. On the other hand, there are also lncRNAs that are not related to PRC2 but equally affect the treatment with bortezomib in MM patients. In this case, in vitro and in vivo approaches showed a better boosted anti-tumor effect for bortezomib in combination with the upregulation of *PRAL* in MM [86]. Likewise, the knockdown of *PDIA3P* and *lnc-ANGPTL1-3* was associated with an increase of sensitivity to bortezomib treatment [60,85], while the upregulation of *DARS-AS1* reduced the sensitivity of MM cells to this drug [41]. Finally, the addition of bortezomib contributes to the upregulation of *XLOC_013703* expression, triggering a decrease in cell proliferation in MM cells [97].

LncRNAs not only interact with PRC2 but also with a great variety of complexes and pathways (Table 1). For example, *TUG1* can be transcriptionally regulated by p53 in response to DNA damage, and can target HDAC4, a histone deacetylase with an oncogenic role in MM [43,94]. *PRAL* also interacts with p53, promoting its upregulation [86,87], while *IRAIN* participates in the regulation of IGF-1 signaling [52]. In the case of *MEG3*, this lncRNA can induce cell apoptosis by both p53-dependent and p53-independent pathways [73]. *DARS-AS1* interacts with HIP-1α, and their inhibition could trigger the suppression of the mTOR pathway [41]. *LUCAT1* (lung cancer associated transcript 1) activates the TGF-β pathway, promoting MM cell proliferation [62]. *HOXB-AS1* (HOXB cluster antisense RNA 1) acts as a scaffold for ELAVL1, modulating the expression of *FUT4*, which could be affecting the Wnt/β-catenin pathway [50]. *MALAT1* acts as a scaffold for PARP1, helping to the repair of damaged DNA (Figure 1) [70]. These results indicate that lncRNAs directly impact on the response of MM patients to the drugs used for their treatment and suggest that their modulation could improve the response rates to the usual treatment schemes used in the therapy of these patients.

## 5. lncRNAs as Biomarkers for Clinical Stratification of MM Patients

Emerging studies have revealed the use of lncRNAs as biomarkers to improve the stratification of patients with different neoplasms, including MM (Table 1) [13,108]. In a recent study we demonstrated that the overexpression of *PDLIM1P4,* or the overexpression of *PDLIM1P4* and *ENSG00000249988*, in combination with clinical and genetic risk factors, divided MM patients into different risk groups, associated with distinct levels of progression-free survival (PFS) and overall survival (OS), respectively [13]. Yin et al. described how high expression levels of *ANRIL* (antisense non-coding RNA in the INK4-ARF locus), combined with the downregulation of *miR-34a*, *miR-125a* or *miR-186*, were associated with worse PFS and OS in MM patients [33]. The overexpression of the cytoplasmic circular lncRNA *Circ_0000190, MEG3* and *PRAL* has been associated with better PFS and OS, and high expression levels of *SMILO* (specific myeloma intergenic long non-coding RNA) with better OS in MM patients [13,36,72,86,87]. By contrast, high expression levels of *lnc-TCF7*, *MALAT1*, *MIAT*, *NEAT1* and *PDLIM1P4* have been associated with worse PFS and OS in MM patients [13,61,63,70,75,76,77,80], the overexpression of *CRNDE*, *LINC00152*, *LINC00461*, *LINC01234*, *lnc-ANGPTL1-3*, *PDIA3P* and *UCA1* with worse OS, and the overexpression of *ENSG00000254343*, *H19* and *NR_046683* (also known as *ST3GAL6-AS1*, ST3GAL6 antisense RNA 1) with worse PFS in MM patients [13,40,46,56,85,93,95]. In addition, high expression levels of *LUCAT1* were associated with worse five-year survival rates [62], and MM patients with the lncRNA *BM742401* methylated showed worse OS than those patients with unmethylated *BM742401* [35]. These results suggest that lncRNAs could significantly contribute to the development of patient stratification tools, improving both the prognosis and monitoring of patients. However, more comprehensive studies are required to put into practice the use of panels of lncRNAs as biomarkers for the stratification of MM patients.

## 6. Epigenetic Drugs can Modulate lncRNA Expression in MM

The rising interest in lncRNAs has led to their consideration as attractive novel therapeutic targets for cancer treatment [109,110]. lncRNAs can form complex interaction networks with chromatin, RNA and proteins, regulating cellular pathways related to cancer hallmarks both directly and indirectly [109]. Therefore, lncRNA-targeted drugs may be an alternative strategy to modulate the activity of well-known oncoproteins, expanding the range of druggable targets for cancer treatment [109,111].

**Figure 2:**
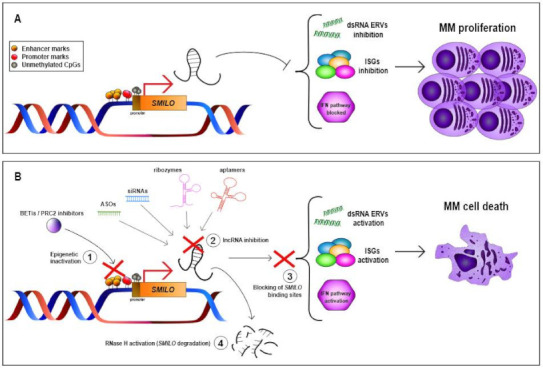
lncRNA-based strategies for MM treatment. (**A**) Gain of de novo active chromatin marks and DNA hypomethylation of the *SMILO* promoter leads to its overexpression in MM cells, triggering the inhibition of ERVs and ISGs and blocking the IFN pathway, resulting in MM cell proliferation. (**B**) Different strategies based on lncRNA-targeted therapies to block the effect of *SMILO* overexpression in MM cells, triggering cellular death. dsRNA = double-stranded RNA; ERVs = endogenous retroviruses; ISGs = interferon-stimulated genes; IFN = interferon; MM = multiple myeloma; BETis = bromodomain and extraterminal inhibitors; PRC2 = polycomb repressive complex 2.

MM is a heterogeneous disease, not only at the genetic but also at the epigenetic level. In fact, the way that the epigenetic changes can deregulate the transcription of lncRNAs has been described [13,73]. For example, recent studies have shown that the downregulation of *MEG3* in MM could be due to DNA hypermethylation of its promoter [72,73,74]. Then, the use of 5-Aza-2′-deoxycytidine could reverse the DNA hypermethylation of *MEG3* promoter, triggering its re-expression and the inhibition of MM cell proliferation [74]. In the case of *BM742401*, this lncRNA was aberrantly DNA-methylated in MM, showing that methylated MM cell lines had lower expression of this lncRNA than unmethylated ones [35]. In contrast, the overexpression of *SMILO* can occur as a consequence of the DNA hypomethylation of its promoter and its transcription from an enhancer region with de novo gain of active chromatin marks in MM (Figure 2) [13]. In this direction, in the last few years, various studies have attempted to describe super-enhancers (SEs), clusters of active enhancers bound to more transcription factor (TF) binding sites than regular enhancers. SEs were first described in MM, where their oncogenic role has been demonstrated [112,113]. Strikingly, SEs could be transcribed into both enhancer lncRNAs (eRNAs) and super-enhancer lncRNAs (SE-lncRNAs), whose deregulation could also affect the development of MM [114]. These examples show the need to develop epigenetic drugs focused on epigenetically altered lncRNAs.

Interestingly, various studies have demonstrated that bromodomain and extraterminal inhibitors (BETis), such as JQ1, can regulate the transcription of enhancer regions or genes regulated by these enhancers [112]. These drugs act specifically on oncogenic SE sites, preventing the binding of their target proteins. BETis increase sensitivity to current therapies for MM, such as those using immunomodulatory drugs (IMiDs), proteasome inhibitors or JAK inhibitors. The combination of those treatments with BETis leads to a further reduction in the expression of oncogenic genes altered in MM, which can result in an increase in cellular apoptosis and anti-myeloma effect [115,116,117]. Specifically, in MM the treatment with JQ1 and lenalidomide triggers the decrease in *MYC* and *IRF4* transcription, leading to reduced MM cell proliferation [113,118,119,120]. Thus, BETis could also be used to inhibit enhancer or SE regions of lncRNAs such as *SMILO* (Figure 2), which is aberrantly transcribed from a de novo enhancer region in MM [13]. In fact, several approaches have demonstrated the effect of these BETis on lncRNAs. For example, it has been described that JQ1 and CPI-203 can reduce the expression of the lncRNA *PVT1* (Pvt1 oncogene) in different MM cell lines [89]. In addition to bromodomains, the PRC2 epigenetic complex is also known to interact with various lncRNAs, leading to the silencing of specific genomic loci. Hence, targeting this interaction could be an interesting approach to modulate downstream epigenetic changes exerted by PRC2 (Figure 2) [110]. In summary, this type of epigenetic therapy could be used to overcome the challenge of treatment resistances and the development of novel strategies against oncogenic lncRNAs in MM.

## 7. lncRNA-Targeted Therapies for MM Treatment

In conjunction with the previously mentioned therapeutic strategies, there are currently several emergent RNA-based strategies that are becoming a major field of research, as they provide an effective way to target altered lncRNAs in certain diseases. RNA-based therapies exploit various effects exerted by oligonucleotides that bind RNA in a sequence-specific manner, which can result in target RNA degradation, alternative splicing redirection, protein production storage or defective RNA repair [121]. Although the majority of these strategies have been developed against coding mRNAs, virtually all RNA species could be targeted equally. By 2020, ten oligonucleotide-based drugs had been approved by the FDA/EMA for the treatment of various genetic diseases, and more than 20 novel compounds are currently being evaluated in clinical trials [122]. However, although a small number of these agents has been selected to target different tumors, preclinical studies have revealed their potential as novel anticancer targeted agents against tumor-specific targets, or even subtype-specific targets [123,124,125].

One of the main strategies involves the knockdown of target deleterious lncRNAs (Figure 2). This can be achieved by specifically designed siRNAs or antisense oligonucleotides (ASOs), which form a heteroduplex with target RNA leading to RNAse H recognition and cleavage. In comparison to conventional siRNAs, ASOs show various advantages, such as higher specificity, reduced off-target effects and RISC complex independence. Alternatively, ribozymes or deoxyribozymes could be used to target lncRNAs that are unfavorable for effective oligonucleotide synthesis due to extensive secondary structures or short length (Figure 2). So far, several studies have successfully attempted to inhibit oncogenic lncRNAs by means of siRNAs or ASOs in the context of cancer treatment (Figure 2) [110]. Regarding MM, some of these oncogenic lncRNAs have been successfully targeted for MM treatment. The most prominent example is the one provided by *MALAT1,* whose druggability has been shown by two independent studies that targeted it with LNA gapmer ASOs both in vitro and in vivo. The degradation of *MALAT1* in vitro, both in cell lines and patient-derived MM plasma cells, induced DNA damage and apoptosis leading to impaired MM proliferation, while in vivo it increased the survival of MM-bearing mice [69,70]. Besides, Hu et al. conjugated the anti-MALAT1 ASOs with single-wall carbon nanotubes (SWCNT), allowing the in vivo release of the drug at high concentrations in MM cells without signals of toxicity in normal cells, which revealed SWCNT as a novel nanomaterial for effective drug delivery [70]. Similarly, a recent study by Taiana et al. showed that *NEAT1* knockdown with ASOs triggered the inhibition of MM cell proliferation and an increase in cellular apoptosis. Furthermore, *NEAT1* inhibition was associated with a chemo-sensitizing effect of both conventional and novel therapeutics, pointing to the interest of targeting *NEAT1* to enhance the effects of novel combination therapies [78].

But oligonucleotide therapies are not only aimed at the inhibition of overexpressed targets. Indeed, splice-switching oligonucleotides may be used to excise exons that encode essential lncRNA functional domains, rendering oncogenic lncRNAs unable to exert their functions. Alternatively, steric blocking oligonucleotides may block the binding of lncRNAs to their binding partners (Figure 2) [109]. Although these strategies have been poorly exploited, they will definitely expand the range of appealing non-coding therapeutic targets for cancer treatment.

However, the development of RNA-based therapeutics still needs to overcome several obstacles if it is going to be translated into clinical practice [110]. In fact, one of the greatest challenges for oligonucleotide therapies is the effective delivery of these agents to target cells. To date, most approved oligonucleotide drugs are focused either on hepatic delivery or local delivery to the eye or the spinal cord, with extra-hepatic delivery remaining a major goal in the field [122]. With the aim of overcoming this obstacle, therapeutic oligonucleotides are chemically modified to improve their drug-like properties. Furthermore, they can be conjugated to a variety of carrier molecules such as aptamers, nanoparticles, antibodies, lipid conjugates, exosomes, peptides or DNA nanostructures, which can promote intracellular uptake, reduce renal clearance from circulation or enhance delivery to the target cells [122]. Among these carriers, aptamers constitute an attractive therapeutic approach due to their unique properties for targeted delivery (Figure 2). These biomolecules combine the flexibility of small molecules with the specificity of antibodies, therefore expanding the range of druggable targets and offering novel solutions to overcome the actual hurdles in targeted delivery [126]. The combination of specific regulatory oligonucleotides with the adequate carriers for selected cells/tissues will enable us to reach previously inaccessible tissues and targets. In this sense, lncRNAs constitute a novel entity of functional transcripts in the cell with demonstrated therapeutic potential. The development of a universal RNA-based therapeutic platform will enable the rapid development of drugs directed to novel target lncRNAs. Therefore, oligonucleotide drugs could be developed against virtually any novel oncogenic lncRNA, undoubtedly expanding the range of druggable targets and leading the way to personalized medicine in tumor treatment in order to respond to unmet clinical needs.

## 8. Conclusions

MM is a heterogeneous disease that needs integrative multidisciplinary studies to shed light on its inter-individual variability. The coding transcriptome of MM is well-characterized, but more high-throughput studies on the lncRNA transcriptome are needed in order to detect all the deregulated lncRNAs that might have an important role in the biology of MM cells, leading to the identification of potential disease biomarkers. Furthermore, functional studies will elucidate their functions and the pathways in which these deregulated lncRNAs are involved, paving the way for the development of novel targeted therapeutic strategies against lncRNAs, as well as for the devising of more effective combination therapies to improve the response and life quality of MM patients.

## Figures and Tables

**Table 1:** Summary of deregulated lncRNAs in MM. MM = multiple myeloma; KD = knockdown; UR = upregulation; up = upregulated; down = downregulated; NA = not available; PFS = progression-free survival; OS = overall survival.

Gene	Location	Gene Type	Expression in MM	Molecular Mechanism	Molecular Interaction in MM	Biological Effect after lncRNA KD	Biological Effect after lncRNA UR	Prognosis in MM	References
***ANRIL***	9p21.3	Antisense	Up	Decoy	Binds to *miR-34a*, *miR-125a*, *miR-186* and *miR-* *411–3p*	Decreases cellular proliferation and increases apoptosis	NA	High expression levels associated with worse PFS and OS	[33,34]
***BM742401***	18q11.2	LincRNA	Down	NA	NA	NA	Decreases cell migration	Methylated lncRNA associated with worse OS	[35]
***Circ_0000190***	1q42.12	Circular lncRNA	Down	NA	NA	NA	NA	High expression levels associated with better PFS and OS	[36,37,38]
***CRNDE***	16q12.2	LincRNA	Up	Decoy	Binds to *miR-451*	Decreases cellular proliferation, increases apoptosis and triggers cell cycle arrest	NA	High expression levels associated with worse OS	[39,40]
***DARS-AS1***	2q21.3	Antisense	Up under hypoxia	Decoy	Interacts with RBM39 and HIP-1α, suppressing mTOR pathway	Decreases cellular proliferation and increases apoptosis. Decreases tumorigenesis in vivo. Its upregulation reduces the sensitivity to bortezomib in vitro	NA	NA	[41]
***ENSG00000249988***	4p15.33	LincRNA	Up	NA	NA	NA	NA	High expression levels associated with worse PFS andbetter OS	[13]
***ENSG00000254343***	8q24.12	LincRNA	Up	NA	NA	NA	NA	High expression levels associated with worse PFS	[13]
***FEZF1-AS1***	7q31.32	Antisense	Up	Decoy	Binds to *miR-610* and regulates AKT3	Decreases cellular proliferation, increases apoptosis and triggers cell cycle arrest	NA	NA	[42]
***GAS5***	1q25.1	Processed transcript	Down	NA	NA	NA	Decreases cellular proliferation	NA	[43]
***H19***	11p15.5	Processed transcript	Up	Decoy	Binds to *miR-152-3p* and *miR-29b-3p*	Decreases cellular proliferation, increases apoptosis and triggers cell cycle arrest	NA	High expression levels associated with worse PFS	[44,45,46]
***HOTAIR***	12q13.31	Antisense	Up	NA	Activates NF-κB pathway	Decreases cellular proliferation, triggers cell cycle arrest and decreases chemoresistance to dexamethasone	NA	NA	[21,47,48,49]
***HOXB-AS1***	17q21.32	Antisense	Up	Scaffold	Scaffold for ELAVL1. Interacts with FUT4-mediated Wnt/β-catenin pathway	Decreases cellular proliferation and increases apoptosis	NA	NA	[50]
***IRAIN***	15q26.3	Antisense	Down	Decoy	Binds to *miR-125b* and regulates IGF-1 signaling	NA	Increases apoptosis	NA	[51,52]
***LINC00152***	2p11.2	LincRNA	Up	Decoy	Binds to *miR-497*	Decreases cellular proliferation, increases apoptosis and triggers cell cycle arrest. Decreases tumorigenesis in vivo	NA	High expression levels associated with worse OS	[53]
***LINC00461***	5q14.3	LincRNA	Up	NA	NA	Decreases cellular proliferation and increases apoptosis	NA	High expression levels associated with worse OS	[54,55,56]
***LINC00515***	21q21.3	LincRNA	Up	Decoy	Binds to *miR-140-5p*	Increases apoptosis	NA	NA	[57]
***LINC00665***	19q13.12	LincRNA	Up	Decoy	Binds to *miR-214-3p*	Decreases cellular proliferation and increases apoptosis	NA	NA	[58]
***LINC01234***	12q24.13	LincRNA	Up	Decoy	Binds to *miR-124-3p*	Decreases cellular proliferation and increases apoptosis. Decreases cell proliferation and tumor growth *in vivo*	NA	High expression levels associated with worse OS	[59]
***lnc-ANGPTL1-3***	1q25.2	Antisense	Up	Decoy	Binds to *miR-30a-3p*	Increases the sensitivity to bortezomib	NA	High expression levels associated with worse OS	[60]
***lnc-TCF7***	5q31.1	NA	Up	NA	NA	NA	NA	High expression levels associated with worse PFS and OS	[61]
***LUCAT1***	5q14.3	LincRNA	Up	NA	Activates the TGF-β signaling pathway	Decreases cellular proliferation, increases apoptosis and triggers cell cycle arrest	NA	High expression levels associated with shorter five-year survival	[62]
***MALAT1***	11q13.1	LincRNA	Up	Decoy and Scaffold	Binds to *miR-1271-5p*, *miR-181a-5p* and *miR-509-* *5p*. Scaffold for PARP1	Decreases cellular proliferation and increases apoptosis	NA	High expression levels associated with worse PFS and OS	[63,64,65,66,67,68,69,70]
***MEG3***	14q32.2	LincRNA	Down	Decoy	Binds to *miR-181a*	NA	Decreases cellular proliferation and increases apoptosis	High expression levels associated with better PFS and OS	[71,72,73,74]
***MIAT***	22q12.1	LincRNA	Up	Decoy	Binds to *miR-29b*	Sensitizes MM cells to bortezomib	NA	High expression levels associated with worse PFS and OS	[75,76]
***NEAT1***	11q13.1	LincRNA	Up	Decoy	Binds to *miR-214* and *miR-125a*	Decreases cellular proliferation	NA	High expression levels associated with worse PFS and OS	[77,78,79,80]
***OIP5-AS1***	15q15.1	Processed transcript	Down	Decoy	Binds to *miR-410* and *miR-27a-3p*	NA	Decreases cellular proliferation and increases apoptosis	NA	[81,82]
***PCAT-1***	8q24.21	LincRNA	Up	Decoy	Binds to *miR-129*	Increases apoptosis and sensitizes MM cells to bortezomib	NA	NA	[83,84]
***PDIA3P***	1q21.1	Pseudogene	Up	NA	NA	Decreases cellular proliferation. Increases the sensitivity to bortezomib	NA	High expression levels associated with worse OS	[85]
***PDLIM1P4***	3q12.1	Pseudogene	Up	NA	NA	NA	NA	High expression levels associated with worse PFS and OS	[13]
***PRAL***	17p13.1	NA	Down	Decoy	Binds to *miR-210*	NA	Decreases cellular proliferation and increases apoptosis. Increases the anti-tumor effect of bortezomib	High expression levels associated with better PFS and OS	[86,87]
***PVT1***	8q24.21	Processed transcript	Up	Decoy	Binds to *miR-203a*. It is inhibited by BRD4	Decreases cellular proliferation and increases apoptosis	NA	NA	[88,89]
***SMILO***	1q42.2	LincRNA	Up	NA	Regulates IFN pathway	Decreases cellular proliferation and increases apoptosis	NA	High expression levels associated with better OS	[13]
***SNHG16***	17q25.1	Processed transcript	Up	Decoy	Binds to *miR-342-3p*	Decreases cellular proliferation, increases apoptosis and triggers cell cycle arrest	NA	NA	[90]
***SOX2OT***	3q26.3	Sense overlapping	Up	Decoy	Binds to *miR-144-3p*	Decreases cellular proliferation, increases apoptosis and triggers cell cycle arrest. Decreases tumor growth *in vivo*	NA	NA	[91]
***ST3GAL6-AS1***	3q12.1	Antisense	Up	NA	NA	Decreases cellular proliferation, increases apoptosis and triggers cell cycle arrest	NA	High expression levels associated with worse PFS	[92,93]
***TUG1***	22q12.2	Antisense	Up	Decoy	Binds to *miR-29b-3p* and targets HDAC4	Decreases cellular proliferation and increases apoptosis	NA	NA	[43,94]
***UCA1***	19p13.12	Processed transcript	Up	Decoy	Binds to *miR-1271-5p* and *miR-331-3p*	Decreases cellular proliferation and increases apoptosis	NA	High expression levels associated with worse OS	[95,96]
***XLOC_013703***	20p11.21	NA	Down	NA	Involved in NF-κB signaling activation	NA	Decreases cellular proliferation and increases apoptosis	NA	[97]
